# The Role of Lactate for Sepsis in Polytrauma Patients, a Time related Analysis using the IBM Watson Trauma Pathway Explorer^®^

**DOI:** 10.26502/jsr.10020268

**Published:** 2022-12-05

**Authors:** Philipp Vetter, Cédric Niggli, Jan Hambrecht, Philipp Niggli, Jindrich Vomela, Richard Chaloupka, Hans-Christoph Pape, Ladislav Mica

**Affiliations:** 1 Department of Trauma Surgery, University Hospital Zurich, 8091 Zurich, Switzerland; 2 Department of Mathematics, ETH Zurich, 8092 Zurich, Switzerland; 3 Division of Medical Sciences in Sportsmedicine, Faculty of Sports Studies, Masaryks University, 62500 Brno, Czech Republic; 4 Department of Orthopedic Surgery, Masaryks University, 62500 Brno, Czech Republic

**Keywords:** WATSON Trauma Pathway Explorer, Artificial intelligence, Lactate, Prediction, Sepsis, Polytrauma

## Abstract

The *Watson Trauma Pathway Explorer*^®^ is an outcome prediction tool invented by the University Hospital of Zurich in collaboration with IBM^®^, representing an artificial intelligence application to predict the most adverse outcome scenarios in polytrauma patients: Systemic Inflammatory Respiratory Syndrome (SIRS), sepsis within 21 days and death within 72 h. The hypothesis was how lactate values woud be associated with the incidence of sepsis. Data from 3653 patients in an internal database, with ongoing implementation, served for analysis. Patients were split in two groups according to sepsis presence, and lactate values were measured at formerly defined time points from admission until 21 days after admission for both groups. Differences between groups were analyzed; time points with lactate as independent predictor for sepsis were identified. The predictive quality of lactate at 2 and 12 h after admission was evaluated. Threshold values between groups at all timepoints were calculated. Lactate levels differed from less than 2 h after admission until the end of the observation period (21 d). Lactate represented an independent predictor for sepsis from 12 to 48 h and 14 d to 21 d after admission relative to ISS levels. AUROC was poor at 2 and 12 h after admission with a slight improvement at the 12 h mark. Lactate levels decreased over time at a range of 2 [mmol/L] for 6–8 h after admission. These insights may allow for time-dependent referencing of lactate levels and anticipation of subsequent sepsis, although further parameters must be considered for a higher predictability.

## Introduction

Accurate triaging plays a pivotal role in patient admission to the trauma bay. For such, various measured and calculated parameters are taken into account to estimate the risk of adverse events (AE) and mortality [1–5]. Recently, the University Hospital of Zurich and IBM collaborated for the construction of an artificial intelligence tool to predict the outcome [Systemic Inflammatory Respiratory Syndrome (SIRS) and sepsis within 21 days, and death within 72h] in polytrauma patients, based on a data sample of more than 3500 patients with ongoing admission [6,7]. The predictive tool is time-related and therefore useful for re-assessment after time periods of up to 21 day after admission. After a previous predictive analysis of the C-reactive protein for sepsis in polytrauma [5], the question arose whether lactate could play a similar role in the course of polytrauma. While lactate increase is commonly caused hypoperfusion [8], it can further indicate an underlying disease process state or adrenergic stress [9–12], e.g. in trauma cases. In this case, cell debris can induce an immunologic-triggered response presenting as SIRS [13]. Further complications include a septic state [14,15] by presence of a confirmed or suspected infection, possibly aggravated by a severe or shock state with additional occurrence of multiple organ damage (MOF), and hypotension [14,15]. Several studies assessed the prognostic value of lactate in septic patients for adverse events (AE) and mortality [16–31]. Specifically, a value greater than 4.0 [mmol/L] was markedly associated with a substantial rate of subsequent death [18,20,28], although the general association seems to be linear [18,25–27] and critically dependent on injury severity [29,30]. As a result, already cases with intermediately elevated lactate levels (2.0 – 3.9 [mmol/L]) based on the actual threshold for hyperlactatemia as > 2 [mmol/L] [19,31] may be at-risk for developing a critical health state or death [17,20–24]. Such heterogeneity of cutoffs values including changes over the posttraumatic course [32–34] complicate the decision-making in septic cases based on this parameter. Therefore, the aim was to analyze the relevance of serum lactate for the prediction of sepsis in a time-dependent manner.

## Materials & Methods

### Patient collective sample

All prospectively enrolled polytrauma patients aged ≥ 16 years with an ISS ≥ 16 [35] were included retrospectively into the data sample. Complete datasets were required. We excluded non-survivors prior to admission and patients referred from external hospitals. The patient cohort was split into 2 groups according to the presence of sepsis within the observational period of 21 days. 3653 patients were included for analysis in *Watson Trauma Pathway Explorer*, based on an ongoing database (2022) introduced on 01.08.1996. In both groups, lactate values were measured at formerly [5] defined time points (admission, 1, 2, 3, 4, 6, 8, 12, 24, and 48 h and 3, 4, 5, 7, 10, 14, and 21 days) after admission to the trauma bay of the University Hospital Zurich.

### Definition of sepsis

Based on the most extreme values in leucocyte count, respiratory rate, heart rate and temperature, the SIRS score was calculated each day [36]. It was calculated for the time frame of hospitalization. Sepsis was defined as a SIRS score ≥ 2 with a focus of infection [14], and had to occur within the observational time frame of 21 days.

### Laboratory analysis

Lactate levels [mmol/L] were measured at the *Institut für Klinische Chemie* at the University of Zurich in a standardized latex-enhanced immune turbidimetry. The same procedure of measurement was applied at each time point.

### Statistical analysis

Patients’ baseline characteristics are presented as means with standard deviations (SD) for numerical variables, as medians with interquartile ranges (IQR) for ordinal data and as percentages for binary variables. An unpaired t-test for numerical variables and a Mood’s median test for ordinal variables served for assessment of differences between the baseline characteristics of the two groups. Differences between groups based on the presence of sepsis were analyzed using the Mann-Whitney-U-Test due to a missing normal distribution according to a Q-Q-plot and an unequal variance. Binary logistic regression served for analyzing lactate an independent prediction factor for sepsis adjusted for ISS, while area under the receiver operating characteristic (AUROC) was assessed to grade the prediction quality of lactate levels. Calculation of threshold values between groups at each timepoint was performed according to the closest top-left threshold method, presenting the threshold point closest to the top-left corner of the ROC plot at each measuring point of lactate levels. R-4.0.2. served for data analysis. The level of significance was set a p < 0.05.

### Ethical approval

This study was conducted according to the guidelines for good clinical practice and the Helsinki guidelines. Research was based on the TRIPOD statement, representing a guideline for multivariable prediction model [37]. Ethical approval for analysis of patient data was granted by the ethical committee of the University Hospital Zurich and the government of Zurich upon the development of the database (Nr. StV: 1–2008) and reapproved for development of the *Watson Trauma Pathway Explorer*^®^ (BASEC 2021–00391).

## 3. Results

### Baseline patient characteristics

3653 patients were included into the data sample, with a mean age of 45.8 ± 20.2 years, 73,4% of patients were male (Table 1). Patients presenting with sepsis had higher values for the (New) Injury Severity Score (NISS /ISS) and the *Acute Physiology And Chronic Health Evaluation* (APACHE)-II-Score than patients without sepsis.

#### 3.1 Differences in Lactate Levels between the Groups:

**Figure 1:**
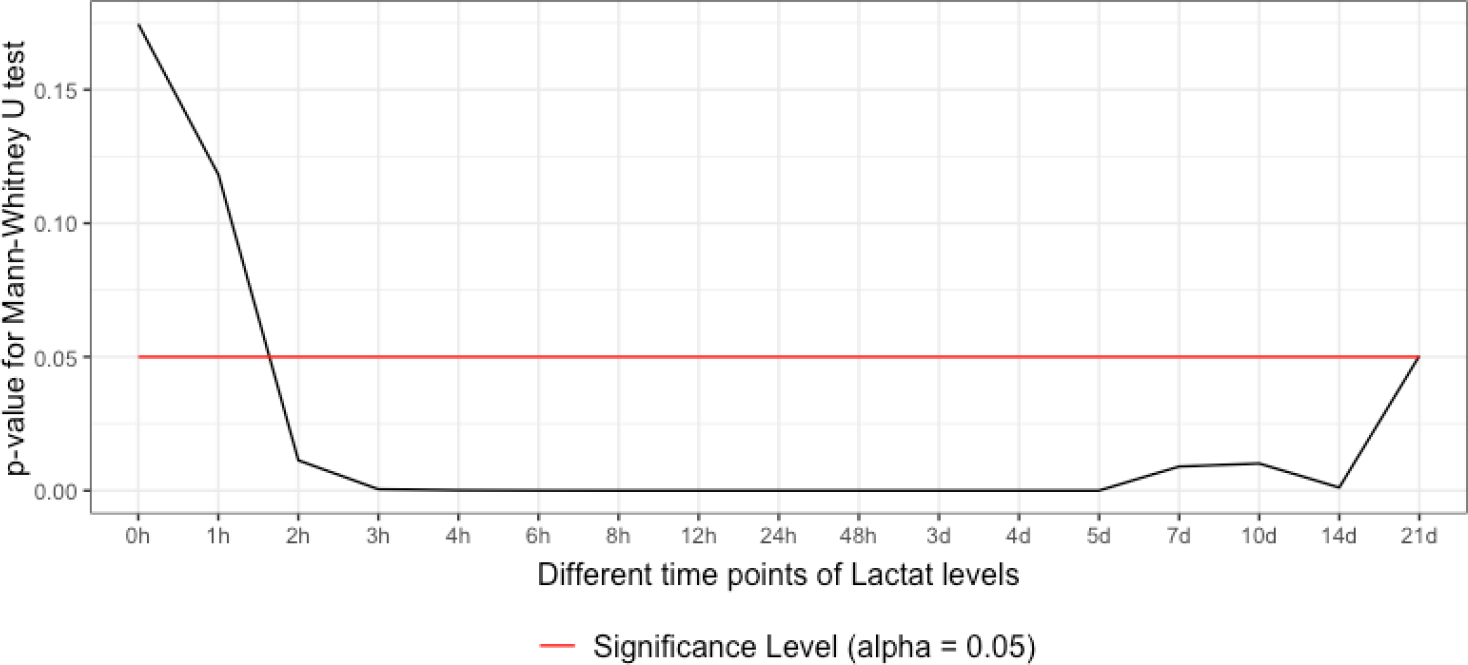
Comparison of lactate levels between the sepsis and non-sepsis group. According to the Mann-Whitney-U-Test, the differences became significant between 1 and 2 h after admission and persisted until the end point of observation.

Testing with the Mann-Whitney-U-Test due to a missing normal distribution, differences in lactate levels between the sepsis and non-sepsis group started to become significant within 1–2 h after admission. The differences continued to be significant over the remaining observational period until 21 d after admission (Figure 1).

#### 3.2 Lactate as an independent predictor for Sepsis:

**Figure 2:**
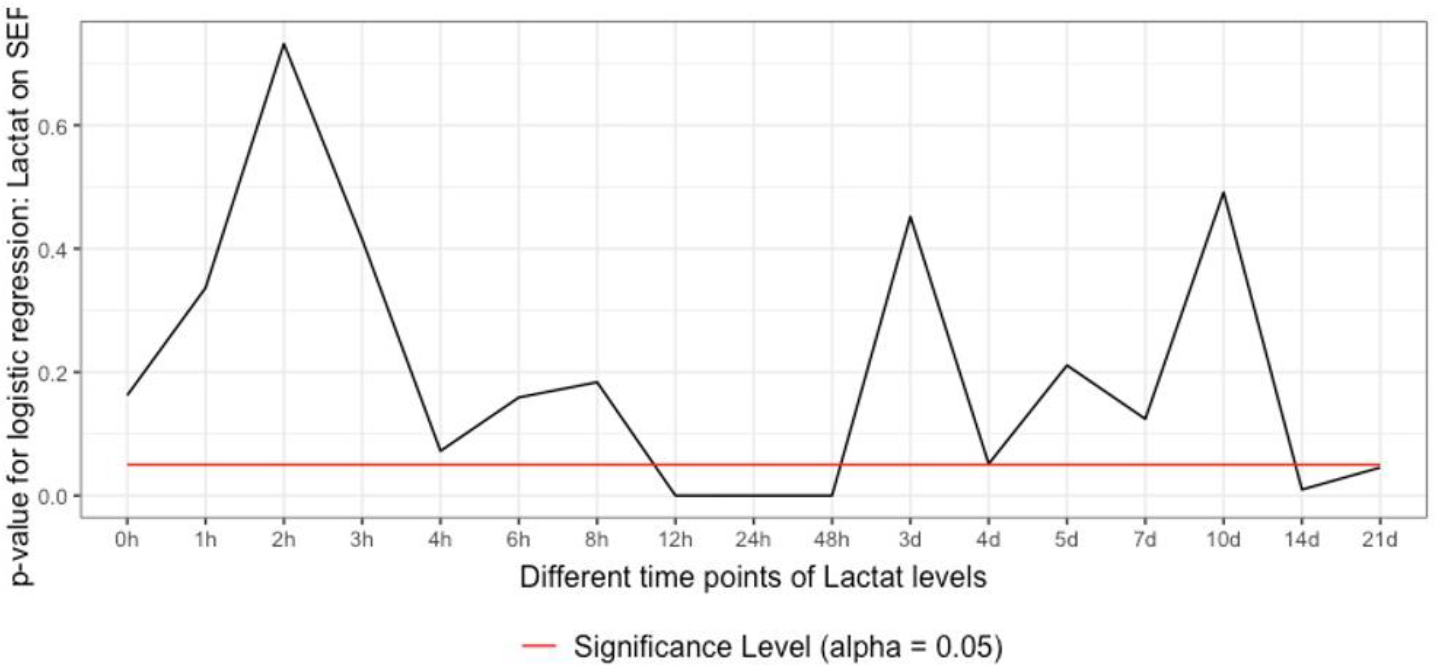
Analysis of lactate in being an independent predictor of sepsis adjusted for ISS. Binary logistic regression revealed an independent predictiveness of between 12 to 48 h and 14 to 21 d after admission.

Binary logistic regression identified lactate as an independent predictor for sepsis in the time frame of 12 to 48 h and 14 to 21 d after admission (Figure 2).

#### 3.3 Prediction quality of lactate for sepsis:

**Figure 3:**
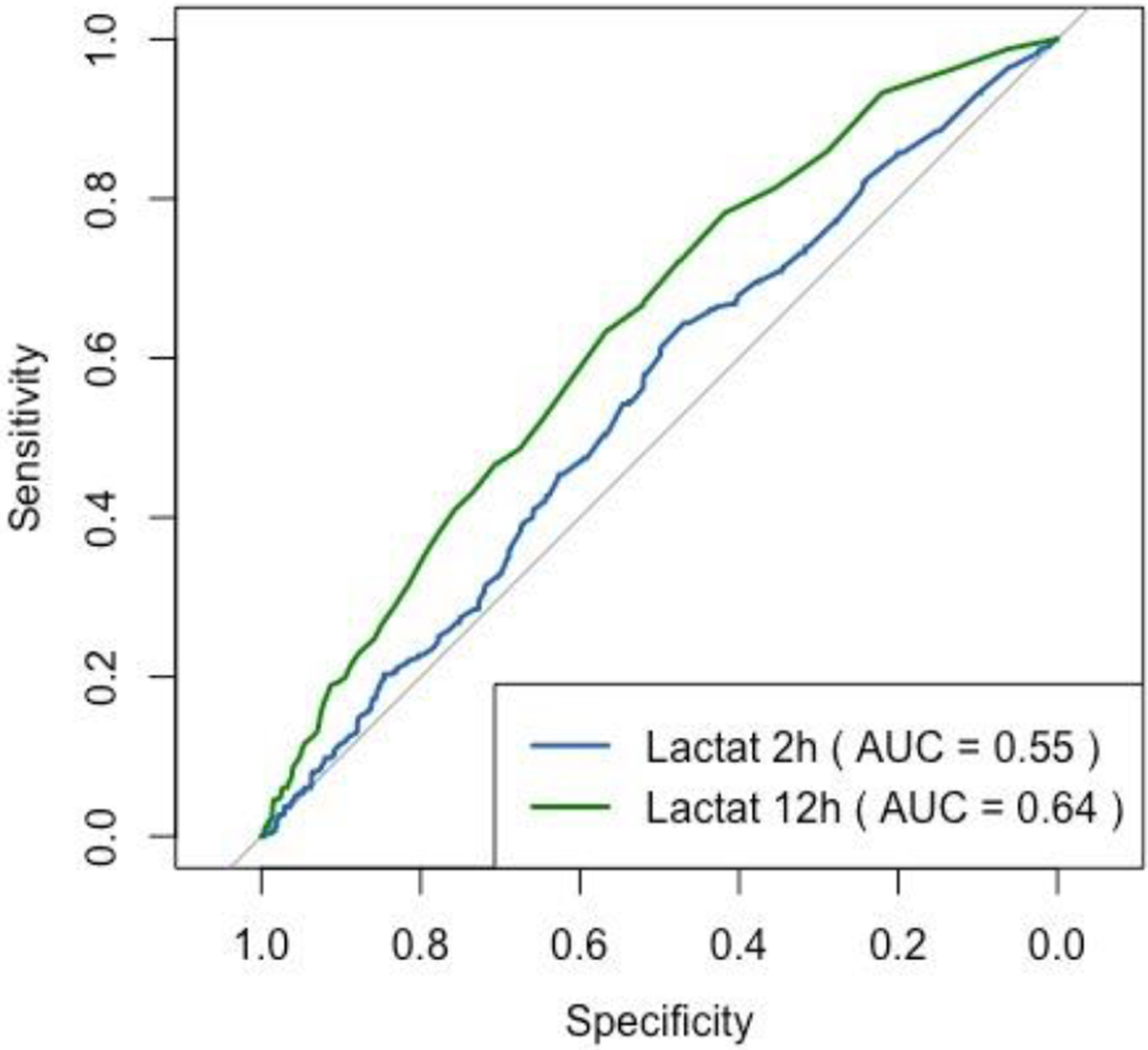
Prediction quality of lactate levels for sepsis. AUROC was slightly higher at 12 h than at 2 h after admission.

The predictive quality of lactate levels was rather poor at 2 and 12 h after admission, with a slightly higher predictability after 12 h (AUROC: 0.64 vs. AUROC 0.55) (Figure 3).

#### 3.4 Time-associated threshold values for sepsis:

**Figure 4:**
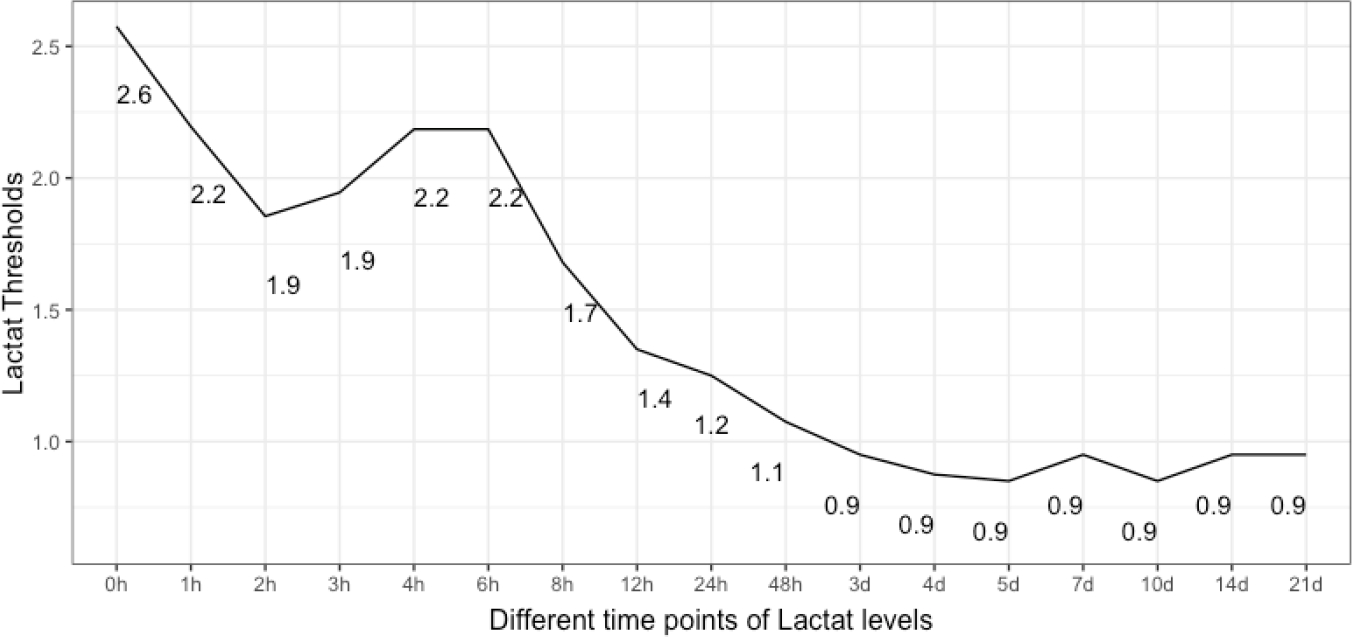
Time associated threshold values of serum lactate concentrations for sepsis. After an initial maximum and a short recurrent gain around 6 h after admission, values decrease asymptotically to physiological levels. Lactate is given as [mmol/L].

The closest top-left threshold method showed a decreasing trend of lactate threshold for sepsis over the observational period (Figure 4). After an initial maximum of 2.6 [mmol/L], levels revolved around 2 [mmol/L] with a slight and short gain around 4 to 6 h after admission before definitely decreasing to physiological values of around 1 [mmol/L] in an asymptotic manner.

## 4. Discussion

The use of the *Watson Trauma Pathway Explorer*^®^ opened the time-associated relevance and threshold of serum lactate for sepsis in polytraumatized patients admitted to our trauma bay, confirming the clinical relevance of intermediately elevated lactate levels [38]. Possible clinical scenarios in polytrauma cases range from SIRS to (severe) sepsis with shock, MOF and death [14,15]. Defining cut-off values of lactate in association with such scenarios is complicated by injury severity, a multifactorial etiology and the course over time [29,30]. In this study, the *Watson Trauma Pathway Explorer*^®^ allowed a time-based risk stratification for the incidence of sepsis in polytrauma patients according to lactate levels. Binary logistic identified lactate as an independent predictor from 12 h to 48 h and 14 d to 21 d after admission relative to ISS levels, confirming it as a a relevant factor in polytrauma patients developing sepsis. The ability to note a difference in lactate between septic and non-septic cases as early as 1.5 h after admission could put the surgeon in an early favorable position and act accordingly by surveillance or intervention. Threshold values for a septic scenario, initially lying within the intermediate range of hyperlactatemia [17,20–24], were identified in a time-dependent manner. Specifically, numbers ranged around the previously reported critical value of 2 [mmol/L] [19,31] for 6 to 8 h after admission before declining definitely. This finding may not only be helpful in the initial assessment, but also over the course of hospitalization. The notion of a lactate decrease over time suggests concordance with the concept of SIRS/CARS (*Compensatory Anti-inflammatory Response Syndrome*), where the initial severe inflammation (SIRS) is calmed resulting in CARS [38]. Clinically, the observations on time-dependent referencing of cut-off lactate levels in the light of sepsis in a polytrauma cohort may facilitate the decision making. This includes administering medication such as fluids, pain relievers, antiobiotics, vasopressors or corticosteroids. From a surgical perspective, lactate may affect the decision for damage-control or early total care according to the risk of an AE by an overshooting immunologic response or an immunosuppression-triggered sepsis. Overall, the lactate values are supposed to serve as a surrogative guidance to minimize complications while providing the most comprehensive treatment. However, as indicated by the AUROC prediction quality, lactate must be seen in a multifactorial process regarding the development of sepsis in polytrauma patients.

There are limitations to be mentioned: Several aspects from patients side were not considered in this study such as chronic diseases including metabolic diseases and respective medication as well as BMI and nutritional status. Previous therapeutic efforts such as admission of antibiotics, fluids or vasopressors were not accounted for. Changes in treatment recommendations since the starting point of data acquisition were not considered. Investigation regarding the known association between lactate levels and mortality [17,18,25,27,28] was not performed. The high rate of early death in non-septic patients could result from airway or breathing impairment in the absence of head injury, complete loss of consciousness or severy bleeding, but specific cause attribution is not possible based on our data analysis. A higher PCT value in non-septic patients at admission should be subject to further research.

## 5. Conclusions

Lactate was higher in septic patients as early as 2 h after admission until the end of the observational period of 21 days. It represented an independent predictor for the development of sepsis from 12 to 48 h and 14 d to 21 d after admission relative to ISS levels. AUROC was poor at 2 and 12 h after admission with a slight improvement at the 12 h mark. The threshold value between groups revolved only slightly above 2 mmol/l before normalizing (< 2mmol/l) within 8 h after admission. Our findings may facilitate the decision making regarding drug application or the extent of surgical procedures being damage-control or total care. Thereby, adverse events by an overshooting immunologic response and an immunosuppression-triggered sepsis are supposed to be minimized while providing the most comprehensive treatment.

## Figures and Tables

**Table 1:** Baseline characteristics of the total patient cohort and groups according to sepsis status. Lactate values are shown from admission onwards. Age [years], temperature [°C], Systolic blood pressure [mmHg], Prothrombin time [seconds], Hemoglobin [g/dL], CRP [mg/L], PCT [ng/mL], Lactate [mmol/L].

Baseline characteristic	Overall patient sample N = 3653	Patients with developed Sepsis N = 547	Patients without developed sepsis N = 3106	p-value
Age (mean, SD)	45.8 ± 20.2	42.8 ± 18.1	46.3 ± 20.5	0.0002
Male	73.4%; N=2681	78.6%; N=430	72.4%; N=2251	-
Early death within 72h	19.3%; N=708	1.46%; N=8	22.5%; N=700	-
Blunt trauma	91.3%; N=3336	94.7%; N=518	90.7%; N=2818	-
Head injury	38.3%; N=1400	44.8%; N=245	37.2%; N=1155	-
BMI at admission (mean, SD)	25 ± 4.4	25.9 ± 4.4	24.8 ± 4.3	<0.001
ISS (median, IQR)	25 (17–34)	30 (25–41)	25 (17–34)	<0.001
NISS (median, IQR)	34 (25–50)	41 (33–50)	34 (24–48)	<0.001
APACHE II at admission (median, IQR)	14 (7–21)	17 (11–21)	13 (6–21)	<0.001
GCS at admission (median, IQR)	10 (3–15)	3 (3–14)	11 (3–15)	<0.001
Temperature at admission (mean ± SD)	35.5 ± 1.7	35.4 ± 1.7	35.6 ± 1.7	0.131
Systolic blood pressure at admission (mean ± SD)	130.7 ± 27.6	128.5 ± 27.7	131.2 ± 27.5	0.0715
Prothrombin time at admission (median ± IQR)	84 (65–97)	80 (61–92)	85 (66–98)	0.1257
Hemoglobin at admission (mean ± SD)	11.4 ± 4	11 ± 2.8	11.5 ± 4.2	0.005
CRP at admission (mean ± SD)	13.74 ± 41.21	23.15 ± 62.96	11.94 ± 35.32	< 0.001
pH at admission (mean ± SD)	7.31 ± 0.13	7.30 ± 0.15	7.32 ± 0.13	0.00632
PCT at admission (mean ± SD)	1.23 ± 4.3	0.48 ± 0.56	1.15 ± 4.86	0.559
Lactate at admission (mean, SD)	2.94 +- 2.53	2.94 +- 2.27	2.94 +- 2.58	0.943
Lactate at 1 hours (mean, SD)	2.76 +- 2.42	2.77 +- 2.15	2.75 +- 2.48	0.941
Lactate at 2 h (mean ± SD)	2.63 ± 2.35	2.95 ± 2.45	2.54 ± 2.32	0.035
Lactate at 3 h (mean ± SD)	2.58 ± 2.25	2.89 ± 2.32	2.50 ± 2.23	0.011
Lactate at 4 h (mean ± SD)	2.51 ± 2.14	2.90 ± 2.33	2.41 ± 2.07	< 0.001
Lactate at 6 h (mean ± SD)	2.33 ± 1.87	2.63 ± 1.85	2.24 ± 1.87	0.00123
Lactate at 8 h (mean ± SD)	2.13 ± 2.44	2.46 ± 1.74	2.05 ± 2.59	0.00851
Lactate at 12 h (mean ± SD)	1.69 ± 1.37	2.10 ± 1.55	1.58 ± 1.30	< 0.001
Lactate at 24 h (mean ± SD)	1.38 ± 1.15	1.71 ± 1.17	1.30 ± 1.22	< 0.001
Lactate at 48 h (mean ± SD)	1.19 ± 1.02	1.47 ± 1.00	1.09 ± 1.01	< 0.001
